# Molecular Imaging with 3′-deoxy-3′[(18)F]-Fluorothymidine (^18^F-FLT) PET/CT for Early Response to Targeted Therapies in Sarcomas: A Pilot Study

**DOI:** 10.3390/diagnostics10030125

**Published:** 2020-02-25

**Authors:** Kalevi Kairemo, Elmer B. Santos, Homer A. Macapinlac, Shreyaskumar Patel, Anthony P. Conley, David S. Hong, Vivek Subbiah

**Affiliations:** 1 Department of Nuclear Medicine, The University of Texas MD Anderson Cancer Center, 1400 Pressler Street, Unit 1483, FCT 16.6005, Houston, TX 77030, USA; Kalevi.Kairemo@gmail.com (K.K.); ebsantos@mdanderson.org (E.B.S.); hmacapinlac@mdanderson.org (H.A.M.); 2 Department of Sarcoma Medical Oncology, Division of Cancer Medicine, The University of Texas, M.D. Anderson Cancer Center, Houston, TX 77030; USA; spatel@mdanderson.org (S.P.); aconley@mdanderson.org (A.P.C.); 3 Department of Investigational Cancer Therapeutics, Division of Cancer Medicine, The University of Texas, M.D. Anderson Cancer Center, Houston, TX 77030, USA; dshong@mdanderson.org

**Keywords:** 18F-FLT PET/CT, sarcoma, targeted therapy, mdm-2, c-met, ^18^F-FDG PET CT

## Abstract

Although 3′-deoxy-3′[(18)F]-fluorothymidine (FLT)-positron emission tomography (PET) has been utilized for tumor response assessment to neoadjuvant chemotherapy in soft tissue sarcomas, it has not been exploited for the assessment of early response to systematically targeted therapies. Herein, we investigated the ^18^F-FLT PET/CT kinetics in patients with sarcoma who received targeted therapies. Among 15 patients with sarcoma who underwent ^18^F-FLT PET/CT, 5 patients (33%) patients were imaged at three time points: At baseline and at 1–15 weeks (*MDM2*-inhibitor treatment), and 10 patients (67%) were imaged twice: At baseline and at 1–4 weeks (*MDM2 inhibitor*, *n* = 5; *c-met* inhibitor *n* = 5). The patients with sarcoma had a total of 18 identifiable tumors. Twelve of 15 patients (80%) demonstrated ^18^F-FLT concentrations changes early, i.e., at 1–4 weeks. Eight patients responded (53.3%), four patients progressed (26.7%) based on FLT change of more than 10% increase, and three patients (20%) demonstrated no change. ^18^F-FLT PET/CT may be used for early response imaging to molecularly targeted therapies in patients with sarcoma. Further larger studies in specific sarcoma sub-types are warranted.

## 1. Introduction

Tumor proliferation and early response to molecularly targeted therapy can be imaged using 3′-deoxy-3′[(18)F]-fluorothymidine positron emission tomography (18 F-FLT PET/CT) [1,2,3,4,5].

Fluorine-18 fluorothymidine (^18^F-FLT) is a structural analog of the DNA component, thymidine; however, it is not incorporated into the DNA. It is entrapped in the cell due to phosphorylation by thymidine kinase, a part of the proliferation pathway. Hence, this is trapped in the cell and accumulates in the cell. The advantage of ^18^F-FLT is that, it is a marker of tumor proliferation and its uptake has been shown to be proportional to the DNA synthesis rate and correlative with proliferative index [1].

Therefore, imaging of cellular proliferation has the potential to become an important diagnostic and/or theranostic tool to not only evaluate the tumor growth rates, but also objectively assess potential response to treatment [1].

^18^F-Fluorodeoxyglucose positron emission tomography/CT (^18^F-FDG PET CT) is an established imaging modality for diagnosis and staging of many types of cancers including various types of sarcomas. In the imaging of bone and soft tissue sarcomas, FDG-PET was reported to be highly sensitive and especially relevant for estimation of individual prognosis. [6,7] ^18^F-FDG PET CT has been used in early response evaluation in patients with Ewing sarcoma to insulin-like growth factor receptor (IGF1R) targeted therapies. However, tumor grading and differentiation from benign versus malignant tumors may be impacted by nonspecific uptake in inflammatory cells and aggressive benign tumors in multiple sarcoma subtypes. Earlier studies of FDG-PET use in sarcoma have demonstrated an overlap between benign tumors and low-grade sarcomas [2,8,9,10].

Given these limitations, other novel imaging methods are warranted. Interestingly, ^18^F-FLT PET/CT has been utilized to image sarcomas and has the potential to be used for grading and staging of multiple aggressive sarcoma sub-types. In a study of ^18^F-FLT PET/CT in the context of neoadjuvant chemotherapy, ^18^F-FLT uptake, but not ^18^F-FDG uptake correlated significantly with tumor grading [10]. Given this background, it would be appealing to non-invasively image cell proliferation and evaluate intra-tumoral kinetics in response to molecularly targeted therapy in sarcoma. We hypothesized that early response and/or resistance to targeted therapy can be evaluated using FLT-PET CT in patients with sarcoma. We evaluated intra-tumoral kinetics by FLT-PET imaging [11], to c-MET inhibitors and MDM2 inhibitors in patients with multiple types of sarcoma.

## 2. Patients and Methods

This was a single-institution study that was approved by the local institutional review board and was compliant with the Health Insurance Portability and Accountability Act. Written informed consent was obtained from each participant. Fifteen patients with histologically proven sarcomas were enrolled. A baseline ^18^F-FLT PET/CT was obtained before treatment with *MDM2* or *C-MET* inhibitor followed by a second ^18^F-FLT PET/CT approximately 1–15 weeks after treatment in all participants (Table 1 and Table 2).

The radiosynthesis of ^18^F-FLT was produced according to a method already described [2,3,4,12]. Briefly, fluorine-18 fluoride was prepared by the ^18^O (p, n) ^18^F reaction using >95% O-18 enriched water as the target material with a titanium window and target holder. The enriched water was bombarded with 17 MeV protons at 30 microamps. At the end of bombardment, the target water was pushed out of the target by helium pressure into the hot cell where the fluorine-18 fluoride was trapped on an anion exchange resin. The ^18^F fluoride was then eluted with [2.2.2.] Kryptofix^®^/potassium carbonate solution and the fluoride was dried by azeotropic distillation with acetonitrile. The ^18^F fluoride was then reacted with the 5′-benzoate of 2,3′-anhydrothymine in DMSO at 160 °C (4); then, after cooling down to 50 °C, 1% NaOH (0.35 mL) heated to 50 °C, 0.2 M NaH_2_PO_4_ (0.75 mL), and 1.5 mL of 8% EtOH/92% 0.01 M NaH_2_PO_4_ were added before passing through an alumina cartridge and loaded on a preparative HPLC column. The FLT was purified by high-performance liquid chromatography on an ^18^C reversed phase column using 8% ethanol/92% 0.01 M NaH_2_PO_4_ as the mobile phase. The yield was typically 20–50 mCi, and the specific activity was 1–4 Ci/mmol.

We reviewed the medical records of patients with advanced cancer including sarcoma who had functional FLT/PET imaging as part of their care at MD Anderson. This study was performed in accordance with the guidelines of the MD Anderson Institutional Review Board (IRB). Because this was a retrospective chart review, IRB waived the consent requirements. They were enrolled on c-MET and mdm-2-based trials available in the institution.

After a washout period from progression of standard care therapy, patients were enrolled in the Phase 1 clinical trials with targeted therapies. Patients had baseline scans that included CT of the chest, abdomen, and pelvis, and a bone scan. This was primarily used for an evaluation of response. FLT studies were performed before the initiation of the study drug, and follow-up images were obtained according to the therapy protocol. Patients fasted for 3 h prior to ^18^F-FLT administration.

PET/CT studies were performed using a Discovery ST8 PET/CT system (GE Healthcare) in combination with the CT component of an 8-MDCT scanner (LightSpeed, GE Healthcare). A single-dose injection of 184–360 MBq (4.96–9.72 mCi) of ^18^F-FLT was administered intravenously. Whole-body PET imaging (WB PET) consisted of 4 or 5 bed positions, 10 min per bed position approximately 60 min after radiotracer injection. PET images were reconstructed using standard vendor-provided reconstruction algorithms. The CT component of the study consisted of a helical scan covering the head to the mid-thighs (120 kVp, 300 mA, 0.5-s rotation; table speed, 13.5 mm/rotation) with no contrast enhancement. Axial CT images were reconstructed with a slice thickness of 3.75 mm. The PET projection data were corrected for random coincidences, scatter, and attenuation. Transaxial images were reconstructed into 128 × 128 pixel images with a pixel size of at least 4.5 mm. PET images were reconstructed using standard vendor-provided reconstruction algorithms that incorporate ordered-subset expectation maximization and were corrected for attenuation using data from the CT component of the examination; emission data were corrected for scatter, random events, and dead-time losses as well using the PET/CT scanner’s standard algorithms. The dose calibrators (CRC-15R; Capintec) were cross-calibrated with the PET/CT measuring instrument to ensure quantitative accuracy of the PET data. Measurements of uptake and retention in tumors were obtained from the WB PET acquisition and compared to normal tissue.

Regional whole-body reconstructed PET/CT data were stored in the Digital Imaging and Communications in Medicine 3.0, part 10, file format, and transferred to a PET/CT image analysis workstation. Three-dimensional volumes of interest (VOIs) of identifiable primary tumors and metastases and source organs were constructed on the CT images, and their positions verified on the corresponding PET images to include all organ activity. These VOIs were then used for PET image analysis. The identifiable source organs analyzed were the heart, liver, gallbladder, kidneys, urinary bladder, small and large intestines, brain, and whole body. Three-dimensional VOI definitions were used to visually inspect for mis-registration due to motion between sequential scans of the same segment. Residual errors were manually corrected by redefining the VOIs when necessary; this was necessary only for the gall bladder and urinary bladder, in the event that gradual accumulation of radioactivity as well as enlargement over the course of the PET scan occurred.

Blinded image interpretation was performed by two experienced nuclear medicine specialists (>15 years of experience each, KK, ES). ^18^F-FLT PET/CT uptake of target lesions was evaluated visually as present or absent. Certainty of the findings was graded on a 3-point scale. Only after the scans were visually interpreted and the final interpretation/score entered for each patient did these specialists determine the maximum SUV for each residual mass/lesion by CT, regardless of whether it was FLT-PET-negative. Percentual FLT changes were calculated as compared to baseline SUV-values, and a change of >10% was considered significant (cut-off, see Discussion).

## 3. Results

A total of 15 patients with sarcoma were included in the study. The results are presented in Table 1 and Table 2. Five patients were treated with an *MDM2* inhibitor. They had a total of seven lesions analyzed with ^18^F-FLT PET CT. The patients had a diagnosis of malignant fibrous histiocytoma, Ewing sarcoma, liposarcoma, Gastro neuroectodermal tumor (GNET), and leiomyosarcoma (Table 1)

The patients underwent FLT scanning at baseline and at least twice after the initiation of therapy. One patient had imaging studies at three time points. The first follow-up study was performed at 2–8 weeks and the second follow up was at 8–15 weeks. The interval between the investigations was at least six weeks. Three of these five patients responded according to the FLT-change, i.e., at least 10% decrease in activity (based on early response criteria). The two patients who did not respond had lung metastases unilaterally or bilaterally.

**Figure 1:**
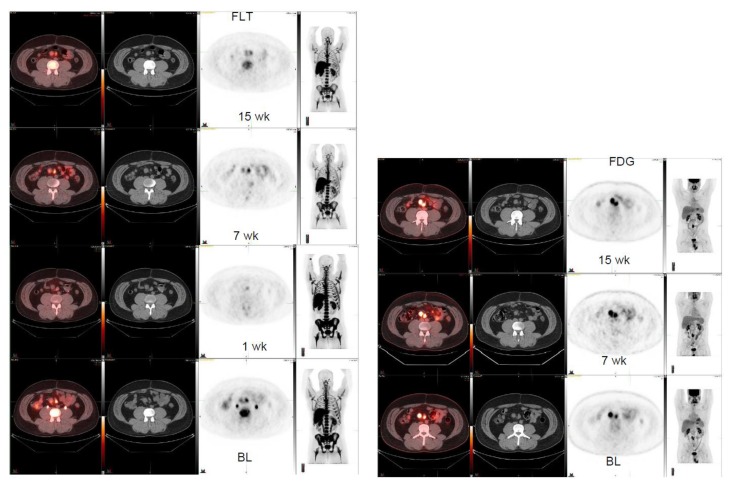
Gastro neuroectodermal tumor (GNET). FLT-study at baseline, at 1, at 7, and at 15 weeks (left panel, 4 rows). In the right mesenteric lymph node, FLT varies: SUVmax 5.2 ->3.2 -> 3.9->2.8 whereas corresponding sizes change on CT as follows: 2.2 cm × 1.9 cm ->2.0 cm × 1.8 cm->2.0 cm × 2.0 cm ->1.9 cm × 1.7 cm. In the left mesenteric lnn, FLT varies: SUVmax 5.5 ->3.4 -> 5.9->4.0 whereas corresponding sizes change on CT as follows: 2.3 cm × 1.6 cm ->1.9 cm × 1.5 cm->2.1 cm × 1.4cm ->2.0 cm × 1.2 cm. FDG-study at baseline, at 7, and at 15 weeks (right panel, 3 rows). In the right mesenteric lnn, FDG varies: SUVmax 12.9 ->nm -> 15.4->10.6 and in the left mesenteric lnn: SUVmax 8.2 ->nm -> 9.9->8.0.

**Figure 2:**
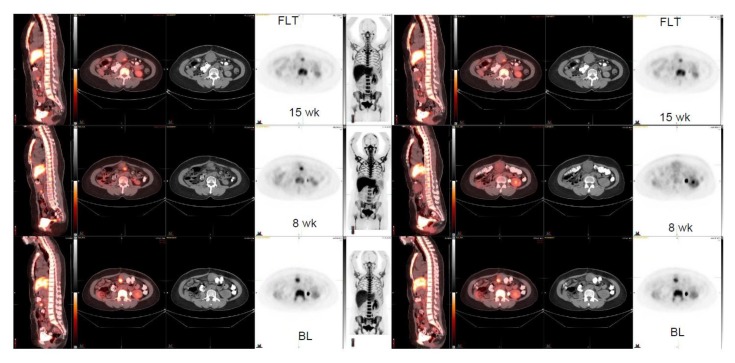
Liposarcoma. FLT-study at baseline, at 8, and at 15 weeks (3 rows). Anterior peritoneal mass is seen; the tumor consists of three components, which are in connection. The biggest tumor changes on CT as follows: 4.1 cm × 3.2 cm ->5.5 cm × 4.1 cm->5.6cm × 5.2 cm. FLT-uptakes changes in the most active site as follows: SUVmax 5.8 ->4.2 -> 3.5.

**Figure 3:**
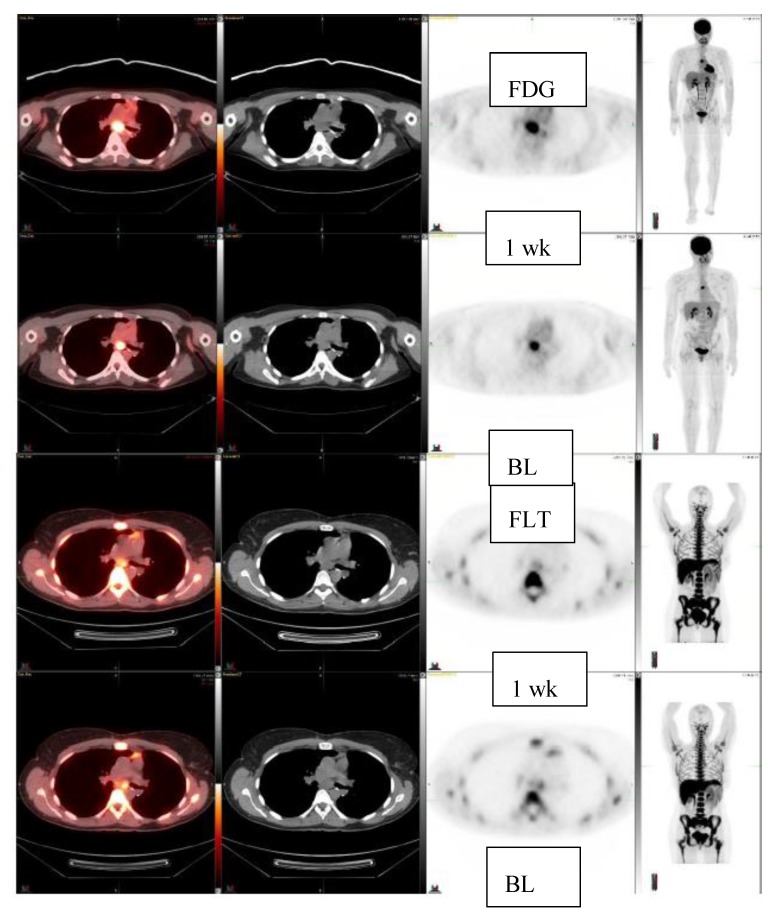
Clear cell sarcoma, left foot, lung resection. FDG-study at baseline and at 1 week (2 upper rows). FLT-study at baseline and at 1 week (2 lower rows). FDG-uptake increases 37% in a subcarinal lymph node: SUVmax 8.9 ->12.2, whereas FLT-uptake decreases 13%, FLT: SUVmax 3.0 ->2.6. On CT it becomes slightly bigger, CT: 2.2 cm × 1.2 cm ->2.3cm × 1.5 cm.

**Figure 4:**
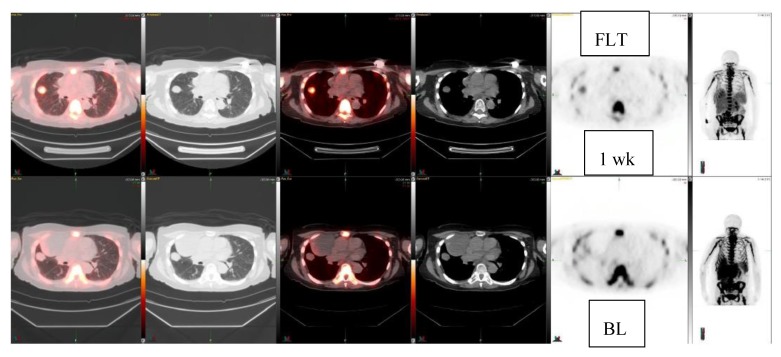
Fibrous tumor, pleura. FLT-study at baseline and at 1 week (2 rows) demonstrates decrease (−43%) in the lung mass: FLT: SUVmax 3.0 ->1.7. On CT it becomes slightly bigger, CT: 2.4 cm × 1.9 cm ->2.5cm × 2.0 cm.

Four patient cases are shown (Figure 1, Figure 2, Figure 3 and Figure 4). A patient with GNET-tumor was studied at baseline and then subsequently at 1, 7, and 15 weeks with ^18^F-FLT. In addition, the patient also had ^18^F-FDG-PET imaging study at baseline and at 7 and 15 weeks (Figure 1). This patient had two mesenteric lymph node metastases (annotated R (right) and L(left)); with ^18^F-FLT, the outcome at seven weeks was -25% (R) and +7% (L), whereas ^18^F-FDG did not show any response (+19% (R) and +21% (L)). Later, at 15 weeks, the response was clear for ^18^F-FLT, with a change of −38% (R) and −38% (L), whereas ^18^F-FDG did not show any clear response (−18% (R) and −2% (L)). A patient with liposarcoma is shown in Figure 2 demonstrating anterior peritoneal mass with three connecting compartments. The patient was studied at baseline and at 1, 8, and 15 weeks with ^18^F-FLT. FLT-uptakes decreased in the most active site as follows: SUVmax 5.8→4.2 (−28%) →3.5 (−40%). The biggest tumor actually increased in size on CT as follows: 4.1 cm × 3.2 cm →5.5 cm × 4.1 cm→5.6cm × 5.2 cm (Figure 2). 

Next, 10 patients who were treated with c-met inhibitor (5 patients) or -2 inhibitor (5 patients) and had a total of 11 lesions were studied for early response with FLT (Table 2). They were studied at baseline and once after the initiation of the new therapy at 1–4 weeks. Five patients demonstrated FLT response, three patients had no change, and two patients progressed. Among these patients, two anecdotal patient cases are detailed in the figures. A patient with clear cell sarcoma of the left foot was studied at baseline and at one week with ^18^F-FLT and with ^18^F-FDG (Figure 3). This patient had a sub-carinal lymph node metastasis. With ^18^F-FLT, the outcome at one week was −13% (R), whereas ^18^F-FDG did not show any response +37%, and SUVmax increased from 8.9 to 12.2. On CT scans, the tumor was slightly larger: 2.2 cm × 1.2 cm →2.3 cm × 1.5 cm (Figure 3). In Figure 4, a patient with a fibrous tumor in pleura is shown, demonstrating a right lung mass. The patient was studied at baseline and at 1 week with ^18^F-FLT and the concentration decreased −43%. On CT, the tumor was slightly larger: 2.4 cm × 1.9 cm →2.5 cm × 2.0 cm (Figure 4).

## 4. Discussion

The utility of ^18^F-FLT in the evaluation of early treatment response in patients with sarcomas is largely unknown, even though several anecdotal non-clinical [13,14,15,16,17] and clinical experience in case reports exist [18]. This is a preliminary report of using ^18^F-FLT-CT in the assessment of response/ early signals of activity for two novel targeted therapies in multiple types of sarcomas.

Sarcomas are very heterogeneous tumors. It has already been shown that diagnosis and staging can be performed in small populations both by using ^18^F-FLT and ^18^F-FDG [2,8,9,10]. Grading between low grade and high grade is also possible with both tracers. In one study [10], mean FLT-SUV in benign lesions was 0.7 (range 0.3–1.3), and 1.3 in low-grade sarcoma (grade1; range 1.0–1.6), 4.1 (range, 2.2–6.0; *p* = 0.002) and 6.1 (range, 2.5–8.3; *p* = 0.001) in grade 2 and grade 3 tumors, respectively. FLT but not FDG uptake correlated significantly with tumor grading (*r* = 0.71 versus *r* = 0.01) [10], and a cutoff value of 2.0 for FLT-SUV discriminated between low- and high-grade tumors. All the tumors in Table 1 were high-grade sarcomas according to the FLT classification and these were imaged three times. The tumors in Table 2 vary from grade 1 to grade 3.

The cut off-value for significant FLT-change has not been discussed in the assessment of early response, because there are no earlier studies nor published criteria. In Figure 3, the decrease of 13% can be seen clearly. The change of 10% being significant is based on our own experience in this material where the highest baseline value was 8.8 and lowest 0.7, respectively. Thus, the 10% change would correspond to SUV changes more than 0.1–0.9 in these patients, which even can be visually observed. There might be a dynamic factor, but 10 of the 15 patients were studied at 1–4 weeks. Typically, PET response is evaluated at 2–3 months. In our case series of only five patients studied for more than three times should be viewed a preliminary. This does not enable further inferences for dynamic behavior of FLT in the early response assessment.

Because thymidine is a DNA base, not found in RNA, which can be labelled with fluorine without essentially changing thymidine kinetics, it acts as a surrogate for DNA synthesis and cellular proliferation. After an intravenous injection, FLT crosses cell membranes and enters tissues by nucleoside transporters, which influence uptake in tumors [4]. This FLT uptake is proportional to cellular proliferation in tumors, whereas glucose uptake is regulated by *glut-1* transporter and characterized by glucose consumption.

Here, we have shown that multiple sarcoma subtypes can be imaged using FLT (Figure 1, Figure 2, Figure 3 and Figure 4. Figure 1 demonstrates that FDG uptake increases early, whereas FLT remains stable or decreases. The FDG uptake may also demonstrate pseudoprogression, which is typical for immunotherapies. Similarly, in Figure 3, the FLT uptake decreases and FDG uptake increases. We also see that the decrease in cell proliferation can be imaged using FLT in spite of the SUV level (Figure 1, Figure 2, Figure 3 and Figure 4).

Although this a small study, we show that FLT may be used to predict responses to c-MET inhibitor or MDM2 inhibitor in diverse sarcoma sub-types. In spite of a very heterogeneous population, we have been able to demonstrate early response in seven out of eight patients who responded according to early response criteria. Early progression based on FLT increase more than 10% was seen in four out of four patients who progressed (Table 1 and Table 2). Early FDG uptake increased in three patients studied at the same time, whereas the FLT uptake decreased. This should be considered as anecdotal, because FDG was not planned for comparison. Additionally, subtle changes and intra-tumor dynamics that may not be readily evaluable in conventional CT scans can be visualized with FLT PETs. Although RECIST responses are used in sarcomas in clinical trials, they have their shortcomings in sarcoma and may also be challenging to interpret (Table 1 and Table 2). Many sarcomas do not shrink when they respond to systemic therapy, especially targeted therapy. Several other criteria have been evaluated in sarcoma to assess response or lack of response to therapies.

FLT PET should always be used as a follow-up of a new therapy for comparison with baseline. A single study may be beneficial only for staging of aggressive tumors with high uptakes. We also observed that intra- and intertumoral heterogeneity could be visualized using FLT, such as in Figure 2. There is a trend of sex difference showing that females’ response is more pronounced than males. However, given the heterogeneity of the tumors, heterogeneity of therapy, different therapies in different dose levels, low number of patients, and the power, it may be premature to make observations or any conclusions based on this data.

In this pilot study, proliferative early response was assessed by ^18^F-FLT-PET scans at baseline and during the course of the trial of c-met inhibitors and mdm2 –inhibitors. Especially, for the 10 patients who were imaged early, all the studies were carried out up to four weeks after initiation of the therapy. The additional five patients, who were assessed in at least three time points up to 15 weeks, were also assessed early. Earlier FLT has been assessed only after completing neoadjuvant therapy. A pilot study by Benz et al., which also studied tissue thymidine kinase 1(TK1) and Ki-67, did not find any correlation between FLT uptake and TK1 nor with Ki-67 [8]. It turned out to be of limited value in the assessment of response; in their 20 patients, 3 responded to neoadjuvant therapy, i.e., mostly chemoradiotherapy or chemotherapy (15 patients totally), whereas targeted therapy was given only to three patients and two patients got radiation only [8].

Here, we tested the use of early FLT in two forms of targeted therapy. In our 15 patient study, change in the FLT behavior was observed in 12 patients, 8 patients demonstrated response, and 4 progressed, meaning that 80% of the patients had new complementary information. There was no change in three patients (20%).

Our study demonstrates that in spite of its heterogeneity and challenges in early diagnostics, FLT may be relevant in the assessment of new targeted therapies in sarcomas.

## Figures and Tables

**Table 1:** The 3′-deoxy-3′[(18)F]-fluorothymidine (FLT)-positron emission tomography (PET) kinetics in a minimum of three time points at 1–15 weeks in patients with sarcoma treated with mdm-2 inhibitors.

Age/Gender	Diagnosis/Site	Baseline SUV(FLT) Size in CT	1st FU SUV(FLT) Size in CT	2nd FU SUV(FLT) Size in CT	Remarks	Outcome (at 7–8 wk)
48/F	Malignant fibrous histiocytoma (MFH), RLL mass	4.1 3.2 × 2.5	3.0 3.5 × 2.8 2 weeks	7.6 5.0 × 3.9 8 weeks		+85%
60/M	Ewing sarcoma, R pelvic mass	3.3 7.9 × 7.0	4.1 7.7 × 7.1 3 weeks	1.9 8.5 × 7.1 10 weeks		−42%
50/F	Liposarcoma, anterior peritoneal mass	5.8 2.6 × 1.8	4.2 2.0 × 1.7 8 weeks	3.5 1.7 × 7.1 15 weeks	Big tumor change 3.2 × 4.1→4.1 × 5.5→ 5.2 × 5.6	−28%
32/ M	GNET, mesenteric lnn R	5.2 2.2 × 1.9 **12.9**	3.9 2.0 × 2.0 **15.4** 7 weeks	2.8 1.9 × 1.7 **10.6** 15 weeks	3.2 (1 week) 2.0 × 1.8 (1 week) **FDG**	−25%+19%
32/ M	GNET, mesenteric lnn L	5.5 2.3 × 1.1 8.2	5.9 2.1 × 1.4 9.9 7 weeks	4.0 2.0 × 1.2 8.0 15 weeks	3.4 (1 week) 1.9 × 1.5 (1 week) FDG	+7% +21%
49/F	Uterine leiomyosarcoma, RLL mass	8.8 4.9 × 3.7	10.7 4.9 × 3.8 2 weeks	11.2 5.2 × 4.2 8 weeks		+27%
49/F	Uterine leiomyosarcoma, LLL mass	8.0 4.2 × 3.6	7.8 4.2 × 3.5 2 weeks	7.6 4.6 × 3.7 8 weeks		−5%

Bold fonts denote SUV_max_ of FDG study.

**Table 2:** The early FLT PET kinetics at 1–4 weeks in patients with sarcoma treated with c-met and mdm-2 inhibitors.

Age/gender	Diagnosis/Site	Baseline SUV(FLT) Size in CT	1st FU SUV(FLT) Size in CT	Remarks	Outcome
42/F	Clear cell sarcoma, Left foot Subcarinal lnn	3.0 2.2 × 1.2	2.6 2.3 × 1.5 1 week		−13%
74/F	Fibrous tumor, pleura Lung mass	3.0 2.4 × 1.9	1.7 2.5 × 2.0 1 week	Fibrous tumor 3.0→1.7 No FDG response	−43%
61/M	Cardiac angiosarcoma, atrium, pericardium	3.3 2.6 × 1.8	3.0 2.0 × 1.7 4 weeks	Another pericardium change 3.9→5.9	+51% -9%
27/F	Alveolar soft part sarcoma, Retroperitoneal implant	1.8 1.1 x1.0	1.6 1.3 x1.0 4 weeks		−11%
54/F	Unclassified high grade sarcoma, distal right thigh musculature	6.2	5.7 4 weeks	Not measurable size on CT	−9%
58/M	Chondomyxoid sarcoma	1.1 22.7 × 17.1	1.4 22.6 × 17.7 1 week		+27%
55/F	Spindle cell sarcoma, Peritoneal implant	0.7 1.2 × 1.0	0.6 1.3 × 1.0 3 weeks		−15%
39/M	High grade sarcoma, RLL mass	7.2 4.8 × 2.7	7.5 4.8 × 2.6 1 week		+4%
47/M	Myxoid liposarcoma, upper mediastinum	2.1 7.4 × 3.8	2.3 7.7 × 4.2 3 weeks		+10%
22/M	Osteosarcoma, right hemithorax, pleura	2.1 3.9 × 3.6	1.6 9.1 × 3.2 4 weeks		−24%
